# Radiological Criteria for Acceptable Alignment in Paediatric Mid-Shaft Forearm Fractures: A Systematic Review

**DOI:** 10.5704/MOJ.2311.005

**Published:** 2023-11

**Authors:** M Scotcher, HH Chong, A Asif, K Kulkarni

**Affiliations:** 1 Department of Plastic Surgery, Addenbrooke's Hospital Cambridge University, Cambridge, United Kingdom; 2 Department of Orthopaedic and Trauma, University Hospitals of Leicester NHS Trust, Leicester, United Kingdom; 3 Division of Surgery and Interventional Science, University College London, London, United Kingdom

**Keywords:** paediatric, forearm, fracture, radiological, outcome

## Abstract

**Introduction:**

Forearm fractures are common in children. The remodelling capacity of growing long bones in children makes these potentially forgiving injuries, recovering with good outcomes despite minimal intervention. Clinicians rely on radiological characteristics that vary with age to guide treatment decisions and minimise adverse sequelae. The purpose of this review was to consolidate the evidence base of radiological indications for intervention in paediatric mid-shaft forearm fractures.

**Materials and methods:**

The preferred reporting items for systematic reviews and meta-analyses (PRISMA) guidelines were followed for this review. Citable research output reporting radiological criteria for mid-shaft forearm fractures in paediatric patients (age ≤16 years) was screened and analysed to ascertain acceptable radiological criteria for non-operative management.

**Results:**

A total of 2,059 papers were initially identified; 14 were selected following screening. Sagittal angulation >15°, coronal angulation >10°, and/or >50% (or >1cm) translation were the most common radiological indications for intervention in children aged 0 to 10 years. For children over 10 years of age, the most common radiological indication for intervention was sagittal angulation >10°, coronal angulation >10°, and/or >50% (or >1cm) translation.

**Conclusion:**

This study revealed a scarcity of high-quality evidence to guide management and significant variation in outcome reporting throughout the published literature. Since Noonan and Price's 1998 recommendations, there has been no significant evolution in the evidence-base guided threshold for intervention in paediatric mid-shaft forearm fractures. There remains a pressing need for a robust multicentre observational study using the patient-reported outcome measurement information system (PROMIS) to address this complex and controversial area of uncertainty in paediatric trauma management.

## Introduction

Paediatric forearm fractures are common injuries, accounting for approximately 32% of all paediatric fractures^1^. Due to the remodelling capacity of growing long bones in children, these have the potential to be forgiving injuries, healing with good outcomes despite minimal intervention. Remodelling capacity is dependent on the age of the child and the site of the fracture; the younger a child from skeletal maturity and the closer the fracture site to the physis, the greater the remodelling capacity of the bone^2^. Simple paediatric distal forearm fractures, therefore, have good healing capacity and are often treated non-operatively with good outcomes^3^.

Diaphyseal (mid-shaft) fractures are less common. Traditionally treated non-operatively, there has been a trend toward operative management of these fractures. This may be due to a combination of fracture alignment and clinical deformity, leaving both family and surgeon concerned about suboptimal clinical outcomes if treated conservatively, alongside evolution in implant technology such as flexible elastic nails and lower profile plates^4^.

Clinicians rely upon age-dependent radiological parameters to guide clinical decision-making and limit complications, with Noonan and Price's 1998 recommendations classically followed for these injuries (Table I)^4^. However, these date back over two decades and are based on yet older studies of malunion, a rare complication of paediatric forearm fracture. With no recent literature review of the validity of these historical recommendations and their correlation with the outcomes, this review seeks to provide evidence-based radiological parameters to guide clinicians in treating these fractures.

**Table I:** Noonan and Price’s 1998 recommendations of acceptable radiological parameters for paediatric forearm fractures

Patient age	Angulation	Rotation	Bayonet apposition
Age 0–9 years (0–8 girls, 0–10 boys)	<15°	<45°	Up to 1cm
Age >9 years (>8 girls, >10 boys)	<10° proximal/midshaft	<30°	Up to 1cm

## Materials and Methods

This review was conducted using the preferred reporting items for systematic reviews and meta-analyses (PRISMA) checklist^5^. The primary outcome of this study was to establish radiological indications for intervention for paediatric mid-shaft forearm fractures, including sagittal and coronal angulation, rotation, and displacement/bayonet apposition.

The electronic databases MEDLINE, EMBASE, CINAHL, and the Cochrane central register of controlled trials were searched from their inception up to Aug 2021, alongside the reference lists of relevant studies (Appendix 1). We included all studies that reported acceptable radiological parameters for non-operative management of single or both bone paediatric (age ≤16 years) mid-shaft forearm fractures. A mid-shaft fracture was defined as occurring in the middle third of the forearm, involving the radius and/or ulna. Proximal and distal third forearm fractures, and injuries with other components (including Monteggia and Galeazzi), were excluded. Exclusions included systematic reviews/meta-analysis, lower quality evidence (including case reports, small (<10 patients) case series, expert opinion), studies of surgical/ diagnostic techniques, cadaveric or biomechanical studies, and foreign language studies for which English language translations could not be obtained.

Three authors independently screened abstracts for the fulfilment of our selection criteria. Full papers were obtained and reviewed if the relevance could not be determined from the abstract. Any disagreement or uncertainty was resolved through discussion with the senior author. All authors independently extracted study characteristics and relevant results from included studies using a standardised data extraction form^6^. We created a narrative of the findings from the included studies, structured around the type of intervention, target population characteristics, radiological parameters, and any reported outcome.

## Results

**Fig 1:**
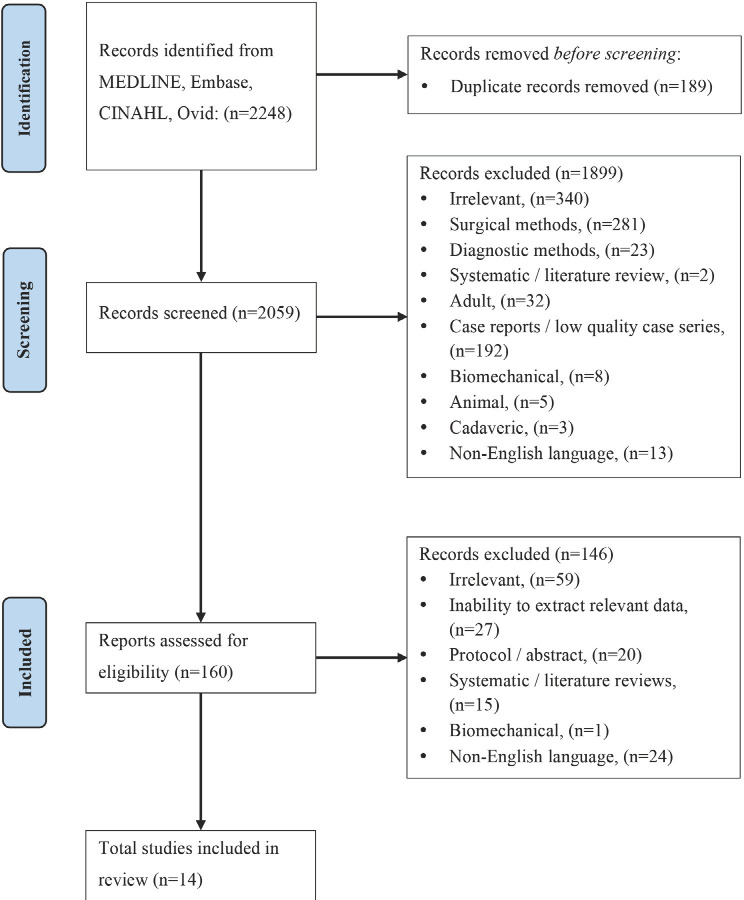
PRISMA flowchart. Exclusion under ‘Irrelevant’ includes non-mid-shaft forearm fracture, no mention of radiological criteria, or only surgical intervention described.

A total of 2,059 papers were initially screened. After exclusions, 160 papers remained for assessment of eligibility. Of these, 14 met our predefined methodological criteria. This is shown in the PRISMA flow diagram below (Fig. 1). The 14 included papers consisted of 10 retrospective studies^7-16^, three prospective studies^17-19^, and one randomised controlled trial (RCT)^20^, with publication dates ranging from 1999 to 2021. Sample sizes ranged from 16 to 431 patients (mean 133, standard deviation SD 109), with a follow-up duration ranging from three weeks to two years. The characteristics of each study are summarised in Table II.

**Table II:** Characteristics of studies

Study / Year	Type of study	Definition of diaphyseal forearm fracture	Sample size (n)	Follow-up duration
Jones *et al* (1999)^7^	Retrospective	Midshaft	431	Not defined
Zionts *et al* (2005)^8^	Retrospective	Diaphyseal	16	49.6 weeks (13-135)
Tarmuzi *et al* (2009)^9^	Retrospective	Diaphyseal - central 3/5	30	> 6 months
Bowman *et al* (2011)^10^	Retrospective	Middle-third	104	Not defined
Sinikumpu *et al* (2012)^11^	Retrospective	Middle-third, diaphyseal	168	Not defined
Iltar *et al* (2013)^18^	Prospective	Diaphyseal	76	Not defined
Colaris *et al* (2013)^20^	RCT	Fracture in the shaft of the bone between distal and proximal metaphysis	127	6 months
Franklin *et al* (2014)^12^	Retrospective	Middle-third	56	>3 weeks
Asadollahi *et al* (2017)^17^	Prospective	Middle-third	269	6-8 weeks
Tasdemir *et al* (2018)^13^	Retrospective	Middle-third	48	Not defined
Kutsikovich *et al* (2018)^14^	Retrospective	Diaphyseal	174	Not defined
Arora *et al* (2018)^19^	Prospective	Midshaft	113	6 weeks
Alagoz *et al* (2020)^15^	Retrospective	Diaphyseal	159	Not defined
Neal *et al* (2020)^16^	Retrospective	Diaphyseal	88	50 days

Abbreviation – RCT: randomised controlled trial

Studies varied in the radiological indication for intervention, particularly in the use of age as a threshold for lower acceptable parameters. In general, for children over the age of nine or ten years, 5 of the 14 studies used a lower acceptable value of sagittal and/or coronal angulation (Table III). While most studies considered sagittal and/or coronal angulation alongside displacement, a few (3 of 14) also considered rotation parameters as indications for intervention, ranging from 0° to 45°^9,10,20^.

**Table III:** Radiological indication for intervention of reported in each study

Study / Year	Radiological Indication for Intervention				
	Sagittal Angulation (°)	Coronal Angulation (°)	Rotation (°)	Displacement / Bayonet	Others
Jones *et al* (1999)^7^	Age <9 : >10	Age <9 : >10		Age <9 : >1cm shortening	
Zionts *et al* (2005)^8^	Age 9-17 : >8 >15	Age 9-17 : >8 >15	-	No apposition	-
Tarmuzi *et al* (2009)^9^	>25	>25	> 0	>1cm	-
Bowman *et al* (2011)^10^	Female age ≤8, Male age ≤10 : >15 Female age >8, Male age >10 : >10	-	>45	-	Single radius fracture, Ulna angulation <15°
Sinikumpu *et al* (2012)^11^	>15	>15		>1cm	-
Iltar *et al* (2013)^18^	Age <7 : >25	Age <7 : >25	-	-	-
	Age 7-9 : >15	Age 7-9 : >15			
	Age >10 : >10	Age >10 : >10			
Colaris *et al* (2013)^20^	Age <10 : >15	-	> 0	> 50%	-
	Age 10-16 : >10				
Franklin *et al* (2014)^12^	-	Ulna: <15°	-	Translation / Shortening	-
Asadollahi *et al* (2017)^17^	>20 dorsal angulation	>10 radial deviation	-	>4mm	Combination of at least 2: >10° of dorsal angulation, >5° of radial deviation, and ≥3mm of translation.
Tasdemir *et al* (2018)^13^	>10	>10	- -	<50% contact	-
Kutsikovich *et al* (2018)^14^	Radius >15	Ulna >10	-	>50% translation	Complete radius fracture
Arora *et al* (2018)^19^	>10	>10	-	Complete displacement	-
Alagoz *et al* (2020)^15^	Age <5 : >25	Age <5 : >25			
	Age 5-9 : >20	Age 5-9 : >20			
	Age >10 : >15	Age >10 : >15			
Neal *et al* (2020)^16^	>15	-	-	Age >10: <50% apposition	-

The most frequently accepted values are shown in Table IV. For children aged between 0 to 10 years of age, the most common radiological indications for intervention were sagittal angulation >15°, coronal angulation >10°, and/or >50% (or >1cm) translation. For children over the age of 10 years, the most common radiological indications for intervention were sagittal angulation >10°, coronal angulation >10°, and/or >50% (or >1cm) translation.

**Table IV:** Most commonly proposed radiological indications for intervention

Patient age	Sagittal Angulation (°)	Coronal Angulation (°)	Rotation	Displacement / Bayonet
Age 0-10 years	>15°	>10°	Limited data	>50% (or >1cm) translation
Age >10 years	>10°	>10°	Limited data	>50% (or >1cm) translation

Only five studies reported clinical outcomes or complications^8,9,14,17,20^. In general, there was only minimal loss of the final range of movement, except in cases of malunion or redisplacement. Overall outcomes were good, albeit notably limited by the widespread lack of reporting. Due to these limitations in outcomes reporting, data pooling and analysis were not possible.

## Discussion

Our findings suggest that the most commonly accepted values for radiological indications for intervention have changed marginally since Noonan and Price in 1998, with the majority in agreement with their recommendation of sagittal angulation >15°, coronal angulation >10°, and/or >50% (or >1cm) translation for children aged between 0-10 years of age; and sagittal angulation >10°, coronal angulation >10°, and/or >50% (or >1cm) translation for children over the age of 10 years^3^. The main difference our study highlights is the important addition of defining both sagittal and coronal angulation. There was insufficient data to comment on rotation as an indication for intervention, with only three studies commenting on this parameter. Two studies^9,20^ suggested any rotation above 0° should receive intervention, while a third suggested any rotation above 45° should be an indication for intervention^10^.

There are many factors for treating clinicians to consider in managing paediatric forearm fractures, including accepting deformity vs. manipulation, stabilising in plaster of Paris vs. modern casting options, placing a below vs. an above elbow cast, what wrist and elbow position to adopt in a cast, whether to wedge the cast to correct deformity and ultimately, whether operative management is indicated. The goal of any management strategy is to maximise outcomes and minimise complications. A fracture is not a uniplanar deformity. There is a need to consider various factors, including the fracture pattern and the impact of all deforming forces on the stability of the individual injury and consequently on the appropriate management^17^. While complications are rare, redisplacement can lead to malunion and poorer functional outcomes. Madhuri *et al's* Cochrane review of non-operative management of paediatric forearm diaphyseal fractures in 2013 highlighted the lack of high-quality evidence for inclusion^21^. Similarly, Abraham *et al's* Cochrane review on operative interventions for these injuries also concluded that no studies were suitable for inclusion^22^. There is, therefore, a need for a robust, prospective study to definitively guide management in this complex area — akin to the ongoing children's radius acute fracture fixation trial (CRAFFT) study, which seeks to answer the same question in distal radius fractures^23^.

Ploegmakers *et al* utilised a combination of the published evidence and expert opinions to construct 'Isala graphs’. These graphical representations plot age (in years) against the acceptable angular deformity (in degrees), with one standard deviation, for various paediatric forearm fractures to guide the intervention threshold^2^. These graphs may be utilised as a part of the decision-making process when considering whether to accept angular deformities in these fractures in patients of different ages. However, the authors also highlighted the heterogeneity of the evidence, focusing on expert opinion and a lack of consideration of the degree of fracture rotation or translation. Ultimately, these graphs were intended to be used as adjuncts to clinical management rather than as definitive treatment protocols.

Measuring and standardising outcomes in paediatric fractures have many challenges, namely achieving active involvement of the child and parent/guardian during a period of stress and pain, and subsequently maintaining longer follow-ups for children who have healed well and are easily lost to review. Crosby *et al* recently attempted to develop a core outcome set for paediatric wrist fractures. They used the following life impact outcomes to measure function: pain intensity, patient satisfaction, ability to return to daily activities, level of difficulty involved in performing everyday tasks, psychological status, and willingness to reuse. They found that although radiological parameters were commonly used as outcome measures, they correlated poorly with patient-reported outcomes^24^. These authors further highlighted the significant heterogeneity in the published literature, and the wide range of outcome measures used, limiting the comparison of interventions between studies. While many outcome domains were similar — namely life impact (ability to function normally), physiological/clinical (range of movement), and technical/surgical considerations (how close the wrist is to normal anatomy) — the measurements used within each domain varied widely and were notoriously difficult to quantify.

This review has several limitations. Firstly, we were limited by inconsistencies throughout the literature in the definition of 'diaphyseal', and for consistency, we adopted a definition of ‘the middle-third of the forearm’. Secondly, the literature reviewed did not consistently separate single bone from double bone fractures or plastic deformity (greenstick) from complete fractures with angulation, which limits this review. Thirdly, we were unable to perform subgroup analysis between the different age groups due to significant heterogeneity in the literature. Finally, the lack of reported short- and long-term outcomes (e.g., range of motion, pain, function, return to activities) limited our ability to make strong clinical recommendations — particularly when an initial malunion in the paediatric population does not necessarily cause an adverse final functional or cosmetic outcome^25^.

We conclude that a high-quality prospective study is required to address this controversy in paediatric mid-shaft forearm fractures. From an ethical standpoint, a randomised controlled trial in a paediatric cohort is potentially inappropriate because it could result in the need for a subsequent osteotomy correction for malunion. We suggest a prospective observational study that is multi-centre or multinational, with homogenous outcome measures using the patient-reported outcomes measurement information system (PROMIS), and the range of motion, radiological outcome, and complications will shed more light on this uncertainty.

## Conclusion

This study has shown no major evolution in the indication threshold for the intervention of paediatric mid-shaft forearm fractures since Noonan and Price's 1998 recommendations. There is marked heterogeneity across the literature and notable inconsistency in outcomes reporting. There remains a pressing need for a high-quality prospective study to address this complex and controversial area of uncertainty in paediatric trauma practice.

## Appendix

**Appendix I:** Search strategy.

1	radius/ or ulna/ or forearm injury/
2	Fracture/ or exp Fracture Fixation/ or exp Fracture healing/
3	and/1-2
4	Radius Fracture/ or Ulna Fracture/
5	((forearm or radius or radial or ulna$1) adj3 fractur$).tw.
6	3 or 4 or 5
7	forearm$1 or midshaft or shaft$1 or diaphys$).tw.
8	6 and 7
9	exp Pediatrics/
10	exp Infant/
11	exp Child/
12	Adolescent/ not exp Adult/
13	(paediatr$ or pediatr$ or neonate$ or bab$3 or infant$ or child$ or teenage$ or adolescen$).tw.
14	or 10 or 11 or 12 or 13
15	8 and 14
